# Vitamin K Epoxide Reductase Complex Subunit 1 (VKORC1) Gene Polymorphisms Predict Arterial Stiffness and Serum MGP Levels in Chronic Kidney Disease Patients

**DOI:** 10.3390/genes16121396

**Published:** 2025-11-21

**Authors:** David H. Chen, Cees Vermeer, John R. Cockcroft, David C. Wheeler, Kevin M. O’Shaughnessy, Yasmin

**Affiliations:** 1 Division of Experimental Medicine & Immunotherapeutics, University of Cambridge, Cambridge CB2 0QQ, UK; d.chen6@ugrad.unimelb.edu.au (D.H.C.); kmo22@medschl.cam.ac.uk (K.M.O.); 2 Cardiovascular Research Institute CARIM, University of Maastricht, 6229 EV Maastricht, The Netherlands; cees.vermeer@outlook.com; 3 Centre for Cardiovascular Research, Innovation and Development, Cardiff Metropolitan University, Cardiff CF5 2YB, UK; jcockcroft@cardiffmet.ac.uk; 4 Department of Medicine, University College London, Centre for Nephrology Rowland Hill Street, London NW3 2PF, UK; d.wheeler@ucl.ac.uk

**Keywords:** arterial stiffness, *VKORC1* gene polymorphisms, vascular calcification, chronic kidney disease

## Abstract

**Background/Objectives:** Arterial stiffness increases with progressive worsening of renal function and predicts cardiovascular mortality in patients with chronic kidney disease. The effects of vitamin K-dependent proteins in vascular health and the implications of vitamin K epoxide reductase gene (VKORC1) polymorphisms in calcification and warfarin sensitivity are well known, but their roles in arterial stiffness are not known. We investigated the influence of common polymorphisms in this gene (−1639G>A, +1173C>T, +1542G>C, +2255C>T, and +3730G>A) on stiffness and calcification markers in 302 CKD patients. **Methods:** Blood pressure, aortic pulse wave velocity (aPWV), coronary artery calcification (CAC), and aortic calcification (AC) were assessed together with the total uncarboxylated matrix Gla protein (t-uncMGP). **Results:** Genotyping subjects for +1542G>C and +3730G>A showed higher genotype-specific aPWV and lower t-uncMGP (*p* < 0.05). The combined recessive allele model showed a significant stepwise reduction in aPWV (*p* < 0.005); subjects homozygous for both risk alleles had the highest aPWV compared to those carrying one or none. In a multiple regression model adjusting for age, gender, mean pressure, BMI, and racial group, each +1542G allele and +3730A allele were independently associated with a 0.8 m/s (95% CI 0.09 to 1.57) and 1.0 m/s (95% CI 0.14 to 1.98) elevation of aPWV, respectively. Although serum t-uncMGP levels correlated inversely with CAC score (*p* < 0.001), VKORC1 genotypes did not. **Conclusions:** We demonstrated for the first time that VKORC1 polymorphisms (+1542G>C and +3730G>A) influence arterial stiffness and serum t-uncMGP levels in CKD patients. These findings suggest that vitamin K-dependent processes may be important in arterial stiffness, possibly by modulating calcification of the vessel wall.

## 1. Introduction

Vascular calcification is the most common complication of chronic kidney disease (CKD), but arterial stiffening is also prominent and is accompanied by the structural and functional changes to the vasculature reflecting tissue damage. Aortic pulse wave velocity (aPWV), a marker of arterial stiffness, increases with progressive worsening of renal function [1]. A number of studies have indicated that increased velocity predicts cardiovascular (CV) mortality in patients with renal disease [2,3,4]. Vascular calcification and arterial stiffness are both heritable conditions that share common risk factors and genetic determinants. Several genes in vascular remodelling, the renin–angiotensin system and extracellular matrix regulation [5,6], and the cytochrome P450 members (CYP2C9 and CYP4F20), which are heme-dependent enzymes involved in xenobiotic metabolism and arachidonate signalling [7], as well as the vitamin K epoxide reductase complex 1 (VKORC1), have been implicated in renal impairment, calcification, and/or arterial stiffening processes. However, the degree of association between these genetic markers and arterial stiffness and/or calcification remains unclear.

Over the past decade, vitamin K-dependent proteins have been shown to play a significant role in cardiovascular health and bone metabolism [8,9,10]. Vitamin K is essential for the carboxylation of vitamin K proteins such as the matrix Gla protein (MGP), which regulates vascular calcification in CKD. Evidence from both animal and molecular studies indicated that vitamin K plays a critical role in the development and progression of vascular calcification, primarily through its involvement in the carboxylation of the matrix Gla protein (MGP) [8], a potent calcification inhibitor expressed in vascular tissue [11,12]. However, findings from human studies remain inconsistent. This variability could be attributed to methodological differences such as variations in sample size, CKD stage, study duration, and analytical techniques, as well as biological heterogeneity and the absence of robust long-term data. Given that VKORC1 is central to γ-glutamyl carboxylation and is inhibited by warfarin, a 4-hydroxycoumarin derivative, it is plausible that the basal VKORC1 enzyme activity may be a determinant of a vascular phenotype. In fact, this was confirmed in wild-derived knockout rats, who had extensive mineralised vasculature compared to control rats [13]. Moreover, potential correlations between various *VKORC1* polymorphisms and indicators of CV mortality have been reported, where several allelic variations within the *VKORC1* gene were associated with arterial calcification and increased cardiovascular disease (CVD) risk [14]. Several studies also reported that *VKORC1* polymorphisms accounted for a major portion of the variance in warfarin dosage [15,16,17,18], making this protein an interesting therapeutic target.

VKORC1 is a 163-amino-acid-long protein with a relative molecular mass of approximately 18 kDa. It is coded by the *VKORC1* gene located on chromosome 16p11.2 and has a number of single-nucleotide polymorphisms (SNPs) in its promoter, non-coding, intronic, and untranslated regions (UTRs) that are in tight linkage disequilibrium (LD). Some SNPs/allelic variations in the promoter (−1639A/G) and intronic (+2255T/C, +1173C/T) regions, as well as five common haplotypes, have been shown to affect the promoter activity, alter VKORC1 mRNA and protein expression [18,19,20], and associate with quantifiable aortic calcification and CVD risk [14]. The data thus far suggests that genetic variants within the *VKORC1* gene affect the risk of vascular calcification in patients with renal disease in several ethnic groups, but to date, their role in arterial stiffness has not been investigated.

**Figure 1:**
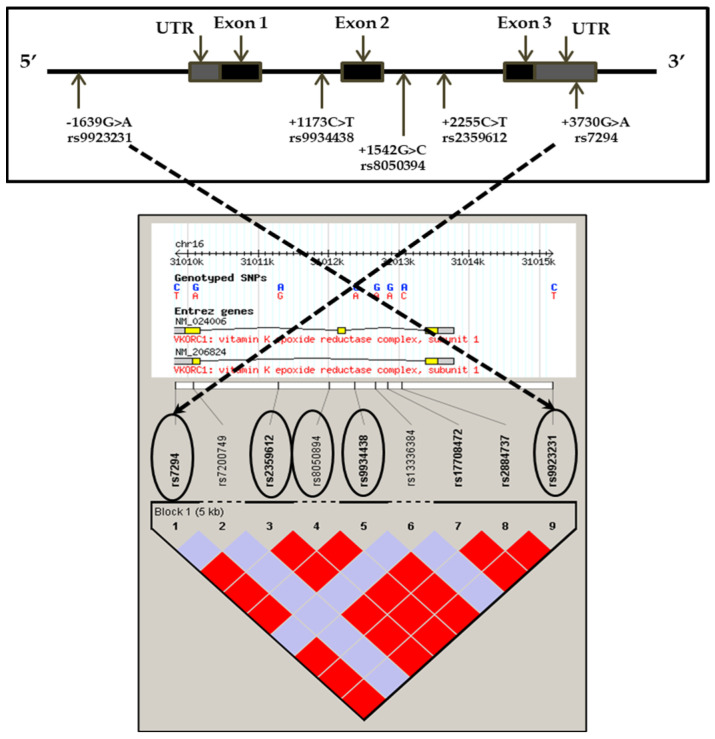
Schematic representation and linkage disequilibrium plot of the *VKORC1* gene showing the five polymorphisms with their exonic, intronic, and UTR positions. The colors represent the relative D′/LOD score where bright red is D′ = 1; LOD ≥ 2 and blue D′ = 1; LOD < 2.

We aimed to investigate the association of the five common *VKORC1* gene polymorphisms, −1639G>A (rs9923321), +1173C>T (rs9934438), +1542G>C (rs8050894), +2255C>T (rs2359612), and +3730G>A (rs7294), with arterial stiffness in CKD patients (Figure 1 illustrates the five SNPs within a 5 kb linkage disequilibrium block and their respective positions). As seen in the figure, one is in the promoter (−1639G>A) region, three are intronic (+1173C>T, +1542G>C, and +2255C>T), and one is located in the 3′UTR (+3730G>A) region. In addition, we also examined their relationships with vascular calcification (coronary artery and aorta) and serum biomarkers (t-uncMGP and eGFR).

## 2. Materials and Methods

### 2.1. Study Subjects and Follow-Up Characteristics

The study group included patients with stages 2–5 chronic kidney disease (n = 302) who participated in the London Arterial Calcification, Kidney And Bone Outcomes (LACKABO) study [21]. The majority of the study participants were predominantly of white ethnicity (73.5%), followed by Asian (12.3%), Black (9.6%), Mixed (4%), and Chinese (1.7%) ethnicities. These patients were selected for their renal disease status based on serum creatinine (greater than or equal to 150 µmol/L for men and greater than or equal to 130 µmol/L for women) to investigate the natural history of calcification.

After a mean of 49 months, the CKD patients were invited for follow-up scans for calcification markers, and just over 50% attended this screening. The reasons for non-attendance could be logistical, health-related hospitalisation, loss of interest over time, or participants misunderstood follow-up requirements. The details of patients re-scanned for measurements are given below.

All studies were conducted in accordance with the Declaration of Helsinki Principles, written consent was obtained from all the study participants, and the study was approved by the Local Research Ethics Committee (MREC Ref No. 03/12/038). Baseline demographic data, height, weight, past and present medical history, and prescribed medications were recorded, and body mass index was calculated.

### 2.2. Determination of Haemodynamic Parameters

All haemodynamic measurements were obtained in a quiet temperature-controlled room. Peripheral blood pressures and heart rates were recorded from the brachial artery of the dominant arm using a validated oscillometric technique (HEM-705CP; Omron Corporation, Kyoto, Japan). Radial artery waveforms were measured from the wrist by a high-fidelity micromanometer (SPC-301; Millar Instruments, Pearland, TX, USA), and the measurements were subsequently used to derive central arterial waveforms through a validated transfer function and technique (SphygmoCor, AtCor Medical, Sydney, Australia). Carotid to femoral pulse wave velocity (PWV), also referred to as aortic PWV (aPWV), was determined from sequential readings of ECG-gated waveforms [22]. aPWV data was available for 199 patients at baseline and 88 at follow-up after excluding poor-quality waveforms and participants with extremely high velocities. All measurements were carried out in duplicate, and the average was used for analysis.

### 2.3. Measurement of Arterial Calcification by Electron Beam Computed Tomography (EBCT)

All CKD patients were screened for calcification status using EBCT, performed at the Royal Brompton Hospital, and using a C-150 scanner (GE-Imatron) with a 100 ms scanning time to take a single 3 mm slice between the carina and the level of the diaphragm. Between 36 and 40 slices were obtained during a single breath hold. Coronary and aortic calcium scores were determined from the computed pixels with a density of >130 Hounsfield units (HUs) and a surface area > 0.51 mm^−2^ [23,24,25,26]. Calcification was later defined by the detection of at least 3 such contiguous pixels. Patients were exposed to an overall radiation dose of 0.9 msv, which is equivalent to approximately one third of the annual exposure from background radiation [27].

Calcification scores were recorded in the aorta and individual coronary arteries such as the left circumflex, left main stem, left anterior descending, and right coronary artery. The total coronary calcification score was determined by averaging the four individual arterial scores, and this value was used in subsequent analysis [24].

### 2.4. Determination of Biochemical Markers

Blood samples were taken in a non-fasting state using standard procedures for the determination of biochemical markers and genetic material. Serum and plasma were separated, and all the samples were then stored at −80 °C. Biochemical and calcification markers, including total cholesterol, high-density lipoprotein, low-density lipoprotein, triglycerides, glucose, creatinine, urea, calcium, and phosphate levels, were determined in an accredited laboratory. The total serum uncarboxylated matrix Gla protein (t-uncMGP) was determined using a competitive (single-antibody) ELISA kit (Biomedica, München, Germany), which was modified via the use of bio-tinylated synthetic uncMGP as a tracer and published previously [28]. The intra-assay and inter-assay coefficients of variation were 5.6 and 9.9%, respectively.

### 2.5. Determination of Genotypes by Taqman System

Genomic DNA (gDNA) was isolated from peripheral venous blood and quantified using standard methods, and genotyping was performed on the ABI Taqman 7500 system (Applied Biosystems, Waltham, MA, USA) in 280 CKD patients where DNA was available. Genotyping was performed after the completion of all clinical and biochemical assessments by an independent research team at Cambridge.

Allelic discrimination was determined by Taqman^®^ Genotyping Assays, each containing two probes labelled by fluorescence. The probes correspond to the alleles harboured by the SNP of interest and carry reporter dyes that are cleaved and released into the solution during PCR by Taq polymerase. The dye is then detected by the assay, and the results are analysed using the accompanying software (version 2.0.4). For each sample, 1 ng of gDNA was added to 13.7 µL of reagent mixture containing Taqman^®^ Genotyping Assays (0.3 µL), Taqman^®^ Genotyping mastermix (7.5 µL), and MQH_2_O (5.9 µL). Aliquots of the final 15 µL reaction were then dispensed into a standard MicroAMP™ Optical 96-well reaction plate and amplified by PCR using the Taqman system. Positive and negative standards were included on each plate. The PCR conditions consisted of pre-PCR reads at 60 °C (1 min) and a holding stage at 95 °C (10 min), followed by 40 PCR cycles at 95 °C (15 s) and a final post-PCR read at 60 °C (1 min). Data were analysed offline with the sequence detection software (version 1.9), and the genotyping success rate was >98%.

### 2.6. Data Analysis

Data were analysed using IBM SPSS Statistics software (version 28.0). All of the variables were checked for normal distribution, and skewed variables were log-transformed appropriately for analysis. Data are expressed as the mean ± SD or SEMs, medians, and ranges, means with 95% CIs, or as percentages, as and where appropriate. Student’s *t*-tests or one-way analyses of variance were used to examine the differences in baseline characteristics, and chi-square tests to calculate genotype frequencies, plus any deviations from Hardy–Weinberg equilibrium (HWE). HWE was assessed prior to conducting any association analyses. Individual haplotypes and their frequencies were estimated using the PLINK programme (version 2.0), and a linkage disequilibrium plot with SNPs was obtained using the Haploview programme (https://www.broadinstitute.org/haploview, accessed on 11 August 2025). Paired-sample *t*-tests were used to check mean differences in calcification and aPWV measurements at baseline and follow-up visits. Statistical analysis by age and gender did not reveal significant differences in baseline characteristics between attendees and non-attendees for calcification scores or aPWV. A correlation analysis was performed to determine associations between clinical and biochemical markers, and a Python programme was used to generate the heatmap. Multiple linear regression analyses were performed to investigate the relationship between normalised aPWV and genetic polymorphisms, as well as to identify independent predictors of aPWV. A probability value of <0.05 was considered significant in the initial analysis. However, to account for multiple comparisons, the Bonferroni correction was applied, and the significance threshold was set to *p* < 0.01.

## 3. Results

### 3.1. Clinical Characteristics

Demographic, clinical, and biochemical data are presented in Table 1 and Table 2. The average age of the cohort was 58 years (range: 19–91 years; Table 1). As expected, 80% of these patients were hypertensive, and over half of them were treated with cardiovascular drugs.

On average, the median coronary artery calcification score was 46.6 (Table 2), and the averages for individual coronary segments were 16.4 for left main stem, 162.1 for left anterior descending, 84.5 for left circumflex, and 148.2 for right coronary arteries. On the other hand, the median aortic calcification score was 52.7.

Of the individuals who attended EBCT screening, 40% had a calcium score of <10. Therefore, the coronary artery calcification scores were divided into the following quartiles as previously reported: 10–100, 100.01–400, 400.01–1000, and >1000.01 [29]. The frequency within each quartile was 18.3%, 18.8%, 10.8%, and 12.1%, respectively. As expected, age and gender were significant determinants of calcification status, and a higher calcification score was also observed in older men (Table 3).

### 3.2. Changes in Calcification Markers over Time

**Figure 2:**
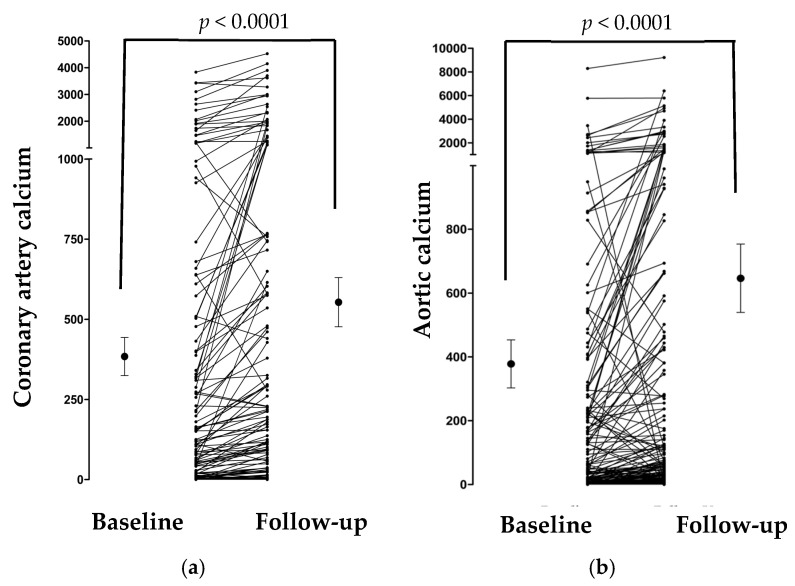
(**a**,**b**) Change in coronary artery and aortic calcification scores at baseline and follow-up visits.

As expected, at 49 months of follow-up, calcification in the coronary artery and aorta increased significantly in these patients (*p* < 0.001, Figure 2a,b; the average mean is presented with 95% CI). However, aPWV reduced marginally (the mean difference was 0.23 m/s; Supplementary Figure S1), which could be related to antihypertensive medications. A similar trend direction was observed for the three markers when patients treated with warfarin were excluded from the analysis.

### 3.3. Genotypes and Haplotypes

The observed genotype distributions of all five SNPs were consistent with the Hardy–Weinberg equilibrium. The minor allele frequencies for each SNP were 35.0% (−1639A), 35.4% (+1173T), 37.4% (+1542C), 35.6% (+2255T), and 41.9% (+3730A) (Supplementary Table S1), which are similar to those reported in other populations. All polymorphisms were tightly linked as shown in Figure 1 (Haploview 2.0).

Results of the haplotype analysis are provided in Supplementary Table S2. Four common haplotypes (those with >5 percent frequency) were identified in this cohort, and the estimated population frequencies are given. Of the four haplotypes identified, the two most common ones (TGGC and CAAT) were significantly associated with aPWV, and this relationship persisted after adjusting for clinically important covariates like age, gender, and BMI (*p* < 0.05).

### 3.4. Differences in aPWV and Calcification Markers for Each SNP

**Figure 3:**
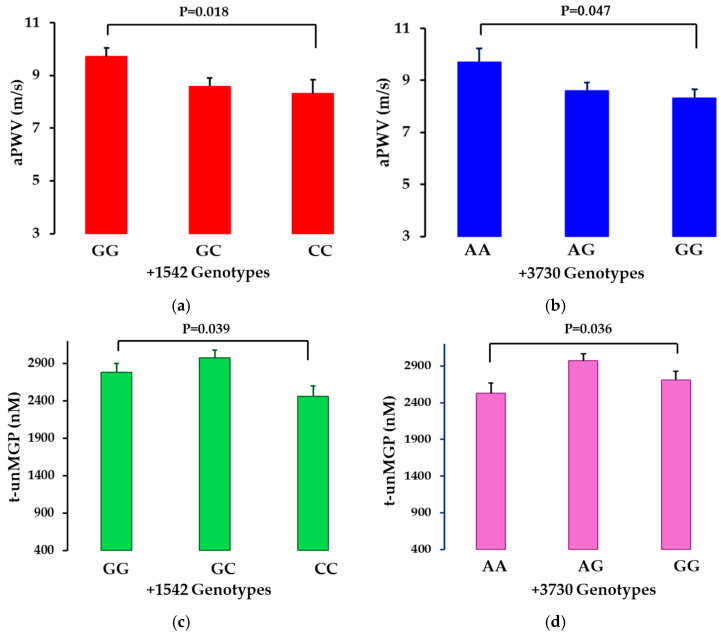
(**a**–**d**) Average aPWV and serum t-uncMGP levels for +1542 and +3730 polymorphisms. Data is shown as arithmetic mean and standard errors.

aPWV and serum t-uncMGP levels were significantly different for the +1542G>C and +3730G>A polymorphisms in the initial analysis (Figure 3a–d). This relationship was clear for aPWV after adjusting for the confounding factors (Table 4). However, the other SNPs did not associate to the same extent with aPWV. Similar differences were found with aPWV for the two SNPs at follow-up, but these differences were not statistically significant due to the small sample size. However, there was no effect of the SNPs on average coronary artery or aortic calcification at baseline or at follow-up.

**Figure 4:**
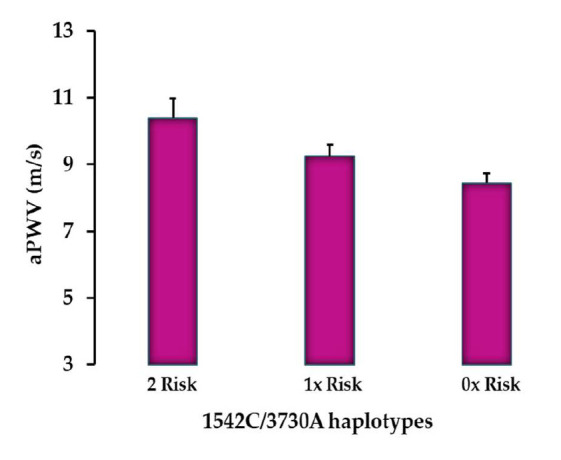
Allelic combination of +1542G>C and +3730G>A polymorphisms predicting a dose-dependent relationship with aPWV. Data shown as arithmetic mean and standard errors.

As variants were tightly linked, a dose-dependent reduction was observed with aPWV (Figure 4). For instance, individuals homozygous for both +1542GG and +3730AA alleles had the highest aPWV values compared to those who were homozygous for only one of the risk alleles at either locus, as well as those who were homozygous for risk alleles at neither locus (*p* < 0.005).

### 3.5. Associations Between aPWV and Each SNP

Since the SNPs that influenced arterial stiffness were tightly linked and the two most common haplotypes were associated with stiffness in this cohort, regression models using recessive alleles were constructed separately for each SNP in order to establish the independent effects of VKORC1 variants on aPWV and t-uncMGP. The +1542G>C and +3730G>A polymorphisms were found to be strong predictors of aPWV after correcting for important covariates (Table 4). Each +1542G allele was independently associated with a 0.8 m/s (95% CI 0.09 to 1.57) higher velocity. And the adjusted R^2^ values observed were modest (~0.28–0.30), indicating that VKORC1 polymorphisms explain only a small proportion of the variability in arterial stiffness, similar to other multifactorial traits like hypertension and diabetes. Similarly, each +3730A allele associated with a 1.0 m/s value (95% CI 0.14 to 1.98) increased the aPWV. When patients on anti-coagulant therapy were excluded from the analysis, the adjusted R^2^ value improved slightly.

### 3.6. Associations Between Age, Haemodynamic, and Calcification Markers

**Figure 5:**
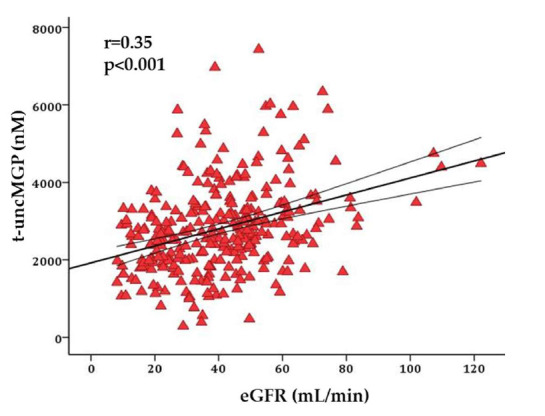
Relationship between t-uncMGP and eGFR levels.

As expected, modest associations were observed between age and calcification markers. Age correlated significantly (*p* < 0.001) with systolic blood pressure, aPWV, calcification scores (CAC and AC), and serum biomarkers (t-uncMGP and eGFR levels), as shown in Supplementary Figure S2. Total coronary calcification score also correlated significantly and inversely with serum t-uncMGP (r = −0.18; *p* = 0.005). Circulating t-uncMGP levels were positively and significantly associated with the kidney function marker, eGFR (r = 0.35; *p* < 0.001; Figure 5).

## 4. Discussion

Arterial stiffness and vascular calcification are important predictors of cardiovascular outcomes in many populations, including CKD patients. However, the molecular mechanisms behind these detrimental processes remain poorly understood. We explored the relationship between VKORC1 polymorphisms, arterial stiffness, vascular calcification scores, and serum t-ucMGP levels in CKD patients. The novel findings were that aPWV was significantly correlated with coronary artery and aortic calcification. Serum t-uncMGP was strongly associated with eGFR, a powerful indicator of renal disease status and calcification scores. Two of the VKORC1 gene polymorphisms significantly affected aPWV and serum MGP levels. These findings suggest that vitamin K-dependent processes may be important in arterial stiffness through previously uncharacterised VKORC1-related mechanisms.

### 4.1. Significant Association Between VKORC1 Gene Polymorphisms and Arterial Stiffness

Many studies validated the usefulness of VKORC1 haplotypes in predicting warfarin dose—specifically, allelic combinations of −1639G>A, +1173C>T, +1542G>C, +2255C>T, and +3730G>A polymorphisms, which account for approximately 30% of the inter-individual variability [30,31]. Several others also explored the impact of +1173C>T and +2255C>T polymorphisms on vascular calcification and cardiovascular outcomes and reported inconsistent results. This discrepancy in the findings may be attributed to population-dependent genetic and environmental factors [9,14,22,23], as linkage patterns and allele frequencies vary widely between biogeographical groups, and this explains most of the inter-ethnic warfarin dose requirements [17,30,31,32].

For the first time, we showed a higher aPWV in carriers of the −1639GG and +3730AA alleles, as did subjects with +1542GG and +2255CC alleles compared to +1542C and +2255T alleles, which is consistent with previous data between the +2255C allele and CVD risk [19]. Of the five polymorphisms, only two (+1542G>C and +3730G>A) were associated with a higher aPWV after adjusting for confounding factors. One explanation could be the location of these SNPs, as well as their possible functional roles. Given that SNPs explored are located in non-coding regions, it is likely that altered VKORC1 expression levels, not structural changes to the protein, are responsible for their functional characteristics. In fact, previous differential mRNA expression work showed that VKORC1 haplotypes predict warfarin dosage [17,31]. So far, only one study demonstrated that the substitution of the second nucleotide in the first E-box (CANNTG) from A to G results in a 44% increase in the promoter activity (−1639G>A) of transfected human HepG2 cells [33]. As E-box sites mediate cell-/tissue-specific gene transcription, this study also showed increased hepatic VKORC1 promoter activity in −1639G allele carriers and, therefore, higher warfarin dose. On the contrary, D’Andrea et al. detected no differences in mRNA splicing as a result of the +1173C>T variant using HELA cells and attributed the predictive potential of this polymorphism on warfarin dosage to its tight linkage with other variants like −1639G>A, which alters VKORC1 activity [13,33].

To date, there is no functional evidence on +1542G>C or +3730G>A polymorphisms, but it is possible that, much like +1173C>T, these polymorphisms serve as tagSNPs for −1639G>A promoter polymorphism, which is consistent with their loci being outside the canonical regions required for exon splicing [17,30,33]. More importantly, the polymorphisms are also conserved across four major species [34], which suggests an evolutionary proof for their putative function in mediating key physiological processes [17,34]. On the other hand, the 3′UTR polymorphism has been shown to alter mRNA expression in other diseases; therefore, the +3730G>A polymorphism may exert its influence on VKORC1 activity in a similar manner [35,36].

In our dataset, +1173C>T, +1542G>C, +2255C>T, and +3730G>A polymorphisms are in tight LD, corroborating previous findings in subjects of European descent. Single SNP and haplotype-based association analyses with confounding factors demonstrated the influence of +1542G>C and +3730G>A on aPWV, confirming that CKD patients have stiffer arteries. This trend was also observed when genotyping information for these two SNPs was pooled. How these variants or other polymorphisms predict high warfarin dosage and/or high transcriptional activity, and eventually lead to stiffer arteries, is unclear and merits further investigation.

### 4.2. No Association Between VKORC1 SNPs and Calcification

Although vitamin K-dependent pathways are involved in both arterial stiffness and vascular calcification, our findings suggest that VKORC1 polymorphisms may have a stronger influence on vascular compliance than calcification in this cohort. This may reflect tissue-specific responsiveness; for example, vascular smooth muscle cells rely heavily on active MGP to inhibit calcification, whereas other vascular beds may respond differently to vitamin K. Additionally, differences in disease progression and biomarker specificity may also contribute to the observed associations. We did not find any association between calcification scores and VKORC1 gene SNPs or the haplotypes, as reported previously [17]. This could be explained by the lack of ability of the EBCT technique to define calcification at a molecular level. For instance, EBCT detects calcified deposits that are formed at more mature stages of calcification, whereas vascular stiffening begins at the microscopic level. Therefore, medial calcification, a major and early contributor to arterial stiffness, cannot be distinguished easily from intima through EBCT. Given that renal disease is polygenic and multifactorial in nature, the effects of the VKORC1 −1639G>A, +2255C>T, and +3730G>A polymorphisms might be less apparent in patients with higher calcification scores. In fact, removing individuals with high-grade calcification in both the aorta and the coronary arteries from the analysis revealed lower *p*-values, which suggests that these SNPs are more informative of vascular stiffness in patients with low-grade calcification. Whether they carry similar implications for individuals with mild or moderately calcified vasculature in other pro-calcifying diseases, such as diabetes, is unclear and merits an investigation.

### 4.3. Association Between VKORC1 Gene Polymorphisms and Serum t-uncMGP

For the first time, we demonstrated an association between some VKORC1 polymorphisms (+1173C>T and +1542C>G) and t-uncMGP levels. Although the combined contributions of the confounders, such as age, gender, mean pressure, BMI, and polymorphisms, were not independently more than 10% as indicated by R^2^ values, it does confirm possible SNP-dependent alterations in gene expression as a factor influencing the activation status of MGP. Specifically, despite the lack of functional evidence, it is possible that +1173C>T, +1542G>C, and +3730G>A exert their effects either via other tightly linked functional polymorphisms or by directly altering mRNA expression levels. Future studies will establish any causal relationship between these variants and serum t-uncMGP levels.

### 4.4. Serum MGP as a Biomarker of Calcification and Arterial Stiffness

Matrix γ-carboxylated Gla protein (MGP) is a vitamin K-dependent protein recognised as a potent inhibitor of vascular calcification. Some studies showed lower levels of serum inactive MGP as an important indicator of severe vascular calcification and poorer outcome in renal disease, while others demonstrated a progressive rise in plasma dephosphorylated–uncarboxylated MGP (dp-ucMGP) levels with CKD stages, aortic calcification scores [37], and aortic stenosis, as well as calcification status in type II diabetes and heart disease [38,39]. In our study, t-uncMGP was inversely correlated with average coronary artery scores and positively correlated with eGFR, reflecting worsening CKD status. A stepwise reduction in t-uncMGP levels was also observed in individuals with increased severity of coronary artery calcification, which is consistent with findings from animal models [11,40]. However, we did not find any association between t-uncMGP and aortic scores, and this could be due to the vascular smooth muscle cell (VSMC) distribution patterns in the larger vessels; coronary arteries have higher VSMC content than elastic arteries such as the aorta. It is, therefore, possible for the aorta to lose the ability to reflect the extent of calcification via MGP synthesis at earlier stages of the calcification process due to VSMC depletion.

### 4.5. Study Limitations

This is a small cross-sectional study exploring the association between *VKORC1* gene polymorphisms and calcification-induced arterial stiffness in patients with CKD. The relatively small sample size at follow-up may reduce statistical power and limit the generalisability of longitudinal findings. Nonetheless, future studies with larger cohorts will shed light on these associations. The observational nature of the study design does not determine the cause–effect relationship between the studied variables, and larger cohort trials are needed to confirm the present findings. Disease states may also affect vitamin K-dependent processes through physiological alterations that are independent of an individual’s genetic profile. It is, therefore, important to consider possible differences in the effects of VKORC1 on vascular stiffness and calcification in cohorts different to the present study.

## 5. Conclusions

For the first time, this study provides clear evidence that the VKORC1 polymorphisms (+1542G>C and +3730G>A) influence arterial stiffness and serum t-uncMGP levels in patients with CKD. The haplotype and allelic combination analyses further confirm a more accurate means of risk stratification. In other words, the number of homozygous risk alleles (+1542GG/+3730AA) carried by an individual was a significant determinant of arterial stiffness. Consequently, several unknown aspects of VKORC1 SNP function and regulatory mechanisms were recognised.

The strong inverse correlation between serum t-ucMGP levels and coronary artery calcification score, as well as the positive association with eGFR, indicates that serum MGP levels are an important biomarker of renal disease status in this cohort. Nevertheless, many questions remain before serum levels of MGP species can be deployed in clinical practice.

## Figures and Tables

**Table 1:** Baseline characteristics of CKD patients.

Variables	Mean ± SD
Age (years)	57.8 ± 15.6
Gender (m/f)	221/81
Height (m)	1.7 ± 0.1
Weight (kg)	81.1 ± 18.8
BMI (kg/m^2^)	27.6 ± 5.5
Current smokers (n)	40
Hypertension (n)	242
Diabetes (n)	61
Myocardial infarction (n)	23
***CVD medications used (n)***	
ACE inhibitor	139
Aspirin	114
α-Blocker	55
α_2_-Blocker	113
β-Blocker	92
Ca^2+^ blockers	102
Diuretics	156
Nitrate	16
Statins	160
Warfarin	12

SD = standard deviation.

**Table 2:** Summary of haemodynamic, calcification, and biochemical markers.

Variables	Mean ± SD
***Arterial Calcification Scores ^a^***	
Total coronary calcification score (baseline, n = 240)	46.6 *(0.0–344.3)*
Total coronary calcification score (follow-up, n = 162)	98.6 *(1.5–587.7)*
Aortic calcification score (baseline, n = 241)	52.7 *(6.2–397.1)*
Aortic calcification score (follow-up, n = 162)	95.6 *(25.4–661.6)*
***Haemodynamic and Arterial Stiffness Measures (n = 302)***	
Peripheral systolic BP, mmHg	133 ± 19
Peripheral diastolic BP, mmHg	79 ± 11
Mean arterial pressure, mmHg	97 ± 12
Heart rate, bpm	69 ± 13
Aortic pulse wave velocity, m/s (baseline, n = 199) ***^a^***	8.5 *(6.8–10.9)*
Aortic pulse wave velocity, m/s (follow-up, n = 88) ***^a^***	7.7 *(5.4–10.6)*
***Biochemical Markers of Vascular Calcification (n = 296)***	
Total cholesterol, mmol/L	4.7 ± 1.1
HDL cholesterol, mmol/L	1.5 ± 0.5
LDL cholesterol, mmol/L	2.4 ± 1.0
Triglyceride, mmol/L	2.5 ± 6.9
Glucose, mmol/L	5.7 ± 2.8
C-reactive protein, mg/L	3.3 ± 4.9
Phosphate, nM	1.3 ± 0.8
Total uncarboxylated matrix Gla protein, nM *^a^*	2670.5 *(2004.0–3359.2)*
Estimated glomerular filtration rate, mL/min *^a^*	39.2 *(25.8–50.9)*

SD = standard deviation; *^a^* = median value with quartile range in *italics*; BP = blood pressure; HDL = high density lipoprotein; LDL = low density lipoprotein.

**Table 3:** Coronary artery calcification scores by age and gender.

Age Groups *(n)*	Men (*n*) *Mean ± SEM*	Women (*n*) *Mean ± SEM*
<40 years *(31)*	4.5 ± 3.0 *(23)*	0.13 ± 0.1 *(8)*
41–50 years *(34)*	89.3 ± 59.3 *(21)*	77.5 ± 51.4 *(13)*
51–60 years *(47)*	425 ± 194 *(33)*	54.6 ± 18.7 *(14)*
61–70 years *(53)*	762 ± 196 *(42)*	75.8 ± 26.3 *(11)*
>71 years *(64)*	875 ± 138 *(50)*	213 ± 130 *(14)*

*SEM = standard error of mean.*

**Table 4:** Determinants of aPWV for each SNP using recessive allele models.

Variables	Beta	t-Value	Significance (*p*)
**−1639G>A polymorphism **			
Age	0.416	6.615	<0.001
Gender	0.031	0.490	0.624
MAP	0.151	2.3096	0.018
BMI	0.227	3.608	<0.001
G>A polymorphism	0.132	2.115	0.036
*Adjusted R^2^ = 0.284; F value = 15.865; p < 0.001*			
**+1173C>T polymorphism**			
Age	0.422	6.724	<0.001
Gender	0.038	0.602	0.548
MAP	0.152	2.417	0.017
BMI	0.229	3.629	<0.001
G>C polymorphism	0.122	1.949	0.053
*Adjusted R^2^ = 0.282; F value = 15.676; p < 0.001*			
**+1542G>C polymorphism**			
Age	0.416	6.582	<0.001
Gender	0.032	0.513	0.609
MAP	0.131	2.081	0.039
BMI	0.247	3.869	<0.001
C>T polymorphism	0.131	2.068	0.040
*Adjusted R^2^ = 0.281; F value = 15.482; p < 0.001*			
**+2255C>T polymorphism**			
Age	0.414	6.542	<0.001
Gender	0.035	0.549	0.584
MAP	0.151	2.405	0.017
BMI	0.230	3.645	<0.001
C>T polymorphism	−0.121	−1.921	0.056
*Adjusted R^2^ = 0.281; F value = 15.646; p < 0.001*			
**+3730G>A polymorphism**			
Age	0.417	6.671	<0.001
Gender	0.016	0.253	0.801
MAP	0.154	2.459	0.015
BMI	0.212	3.367	<0.001
G>A polymorphism	0.159	2.543	0.012
*Adjusted R^2^ = 0.292; F value = 16.345; p < 0.001.*			

## Data Availability

The original contributions presented in this study are included in the article/Supplementary Material. Further inquiries can be directed to the corresponding author.

## Supplementary Materials

The following supporting information can be downloaded at: https://www.mdpi.com/article/10.3390/genes16121396/s1. Table S1: Genotype and allele frequencies. Table S2: VKORC1 haplotype frequencies and their effect on aPWV. VKORC1—vitamin K epoxide reductase complex 1; aPWV—aortic pulse wave velocity. Table S3: Genotype differences in average aPWV, calcification scores, and serum t-uncMGP. aPWV—aortic pulse wave velocity; t-uncMGP—total uncarboxylated matrix Gla protein. Figure S1: Change in aPWV at baseline and follow-up visits. *—average mean is presented with 95% CI; aPWV—aortic pulse wave velocity; MAP—mean arterial pressure; BMI—body mass index. Figure S2: Correlation matrix heatmap. BMI—body mass index; SBP—systolic blood pressure; DBP—diastolic blood pressure; MAP—mean arterial pressure; aPWV—aortic pulse wave velocity; CAC—coronary artery calcification; AC—aortic calcification; t-uncMGP—total uncarboxylated matrix Gla protein; eGFR—estimated glomerular filtration rate.
