# Supplementary material for: Vitamin K Epoxide Reductase Complex Subunit 1 (VKORC1) Gene Polymorphisms Predict Arterial Stiffness and Serum MGP Levels in Chronic Kidney Disease Patients
Source: Genes (Basel). 2025 Nov 21;16(12):1396. doi: 10.3390/genes16121396 (PMC12733207; doi:10.3390/genes16121396)

Supplementary Table S1. Genotype and allele frequencies.

**Genotypes**	**N (%)**	**Alleles**	**N (%)**
-1639G>A polymorphism			
GG	128 (*45.7*)	G	364 *(65.0)*
AA	44 (*15.7*)	A	196 *(35.0)*
AG	108 (*38.6*)		
+1173C>T polymorphism			
CC	126 (*45.0*)	C	362 *(64.6)*
TT	44 (*15.7*)	T	198 *(35.4)*
CT	110 (*39.3*)		
+1542G>C polymorphism			
CC	118 (*42.4*)	C	348 *(62.6)*
GG	48 (*17.3*)	G	208 *(37.4)*
GC	112 (*40.3*)		
+2255C>T polymorphism			
CC	125 (*44.6*)	C	360 *(64.3)*
TT	45 (*16.1*)	T	200 *(35.6)*
CT	110 (*39.3*)		
+3730G>A polymorphism			
GG	102 (36.6)	G	324 *(58.1)*
AA	57 (*20.4*)	A	234 *(41.9)*
AG	120 (*43.0*)		

Genotype percentages are given in parenthesis in *italics.*

Supplementary Table S2. VKORC1 haplotype frequencies and their effect on aPWV.

Haplotype Sequence	Frequency of haplotypes	Haplotype effect on aPWV (P-value)
TGGC	0.395	<0.001
CAAT	0.338	<0.005
CGGC	0.238	<0.05
CAGC	0.009	-
TAAT	0.005	-
CGAT	0.004	-
CAGT	0.004	-
TGAC	0.004	-
TAGC	0.002	-
CAAC	0.002	-

Supplementary Table S3. Genotype differences in aPWV, calcification scores and serum t-uncMGP at baseline.

	**aPWV**<br><br>***Mean ± SD***	**Average coronary calcification score** [a]<br>***Mean ± SD***	**Average aortic calcification score** [a]<br>***Mean ± SD***	**Serum t-uncMGP** [a]<br>***Mean ± SD***
-1639G>A polymorphism				
GG	9.0 ± 1.4	114.1 ± 7.6	85.1 ± 9.5	2254.1 ± 1.6
AA	8.3 ± 1.4	116.2 ± 7.2	62.3 ± 9.5	2244.4 ± 1.6
AG	8.1 ± 1.4	148.4 ± 10.7	128.8 ± 9.5	2685.8 ± 1.5
P=	0.126	0.772	0.290	0.098
+1173C>T polymorphism				
CC	8.9 ± 1.4	123.3 ± 7.6	141.3 ± 9.6	2547.5 ± 1.6
TT	8.3 ± 1.4	116.2 ± 7.7	62.3 ± 11.5	2244.7 ± 1.6
CT	8.2 ± 1.4	134. 2± 10.6	77.8 ± 9.3	2682.5 ± 1.5
P=	0.280	0.956	0.140	0.101
+1542G>C polymorphism				
GG	9.2 ± 1.3	120.8 ± 7.6	82.8 ±8.8	2474.4 ± 1.7
CC	7.82 ± 1.4	110.4 ± 9.3	73.6 ± 11.5	2223.9 ± 1.6
GC	8.1 ± 1.4	134.8 ± 9.6	115.8 ±9.9	2784.2 ± 1.4
P=	**0.007**	0.919	0.532	**0.012**
+2255C>T polymorphism				
CC	9.0 ± 1.4	117.8 ± 7.7	85.9 ± 9.7	2547.4 ± 1.6
TT	8.3 ± 1.4	116.2 ± 7.2	62.3 ± 11.5	2286.6 ± 1.7
CT	8.2± 1.4	142. 0± 10.5	126.7 ± 9.4	2665.5 ± 1.5
P=	0.151	0.869	0.308	0.178
+3730G>A polymorphism				
GG	8.2 ± 1.4	136.8 ± 8.3	81.2 ± 12.0	2477.7 ± 1.6
AA	9.6± 1.4	113.0 ± 7.8	57.2 ± 8.1	2290.5 ± 1.7
AG	8.5 ± 1.4	125.2 ± 9.3	143.6 ± 8.7	2752.2 ± 1.5
P=	**0.047**	0.921	0.080	**0.036**

[a] Values given are geometric mean ± geometric standard deviation (GSD)

Supplementary Figure S1. Change in aPWV at baseline and follow-up visits.

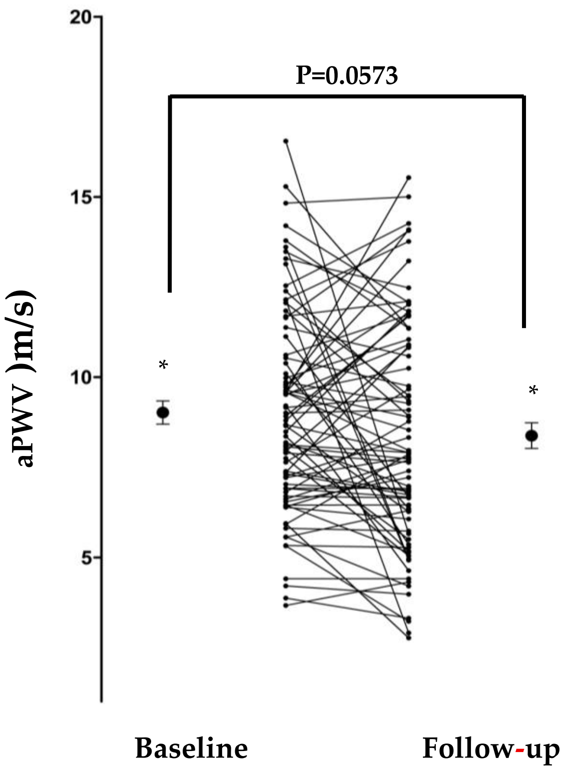

* Average mean is presented with 95% CI.

Supplementary Figure S2. Correlation matrix heatmap.

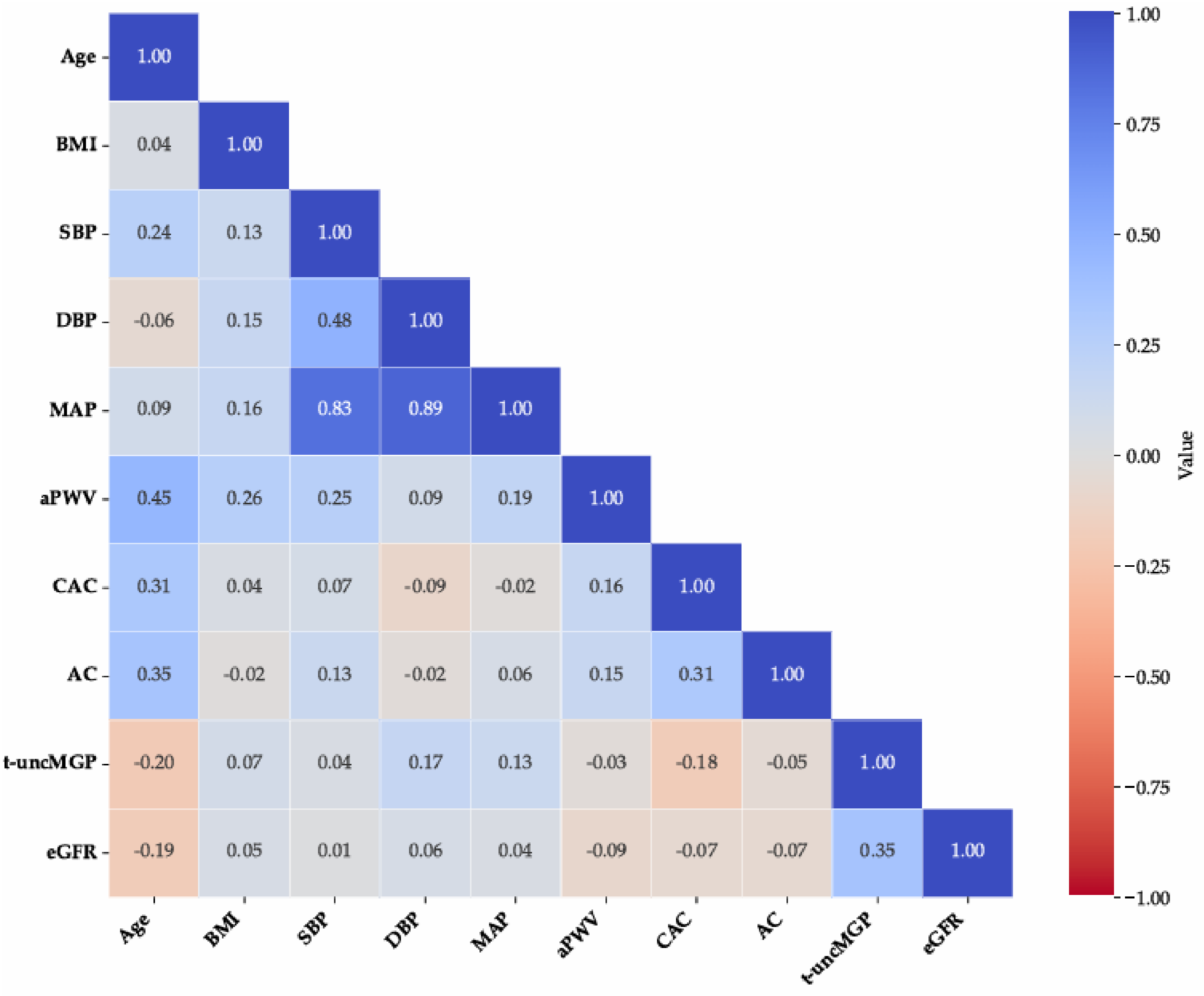

Supplement: Supplementary file 1 [file genes-16-01396-s001.zip › Genes-3869960-Supplementary.pdf]
